# Productive integration, economic recession and employment in Europe: an assessment based on vertically integrated sectors

**DOI:** 10.1007/s40812-020-00178-3

**Published:** 2020-12-24

**Authors:** Davide Villani, Marta Fana

**Affiliations:** 1 The Open University (UK), Goldsmiths College, University of London, Milton Keynes, London, England; 2 Joint Research Centre, European Commission, Seville, Spain

**Keywords:** Vertically integrated labour, Covid-19, Global value chains, Productive integration, C67, J21, L16

## Abstract

The Covid-19 crisis has revamped the discussion about the redefinition of GVC. This paper contributes to the debate, analysing the productive relationships between European countries in four key manufacturing activities. In particular, the paper addresses two objectives. First, it maps the degree of productive integration in Europe, focusing on the generation of employment in the production of exported intermediate inputs and final goods. Second, it provides a preliminary assessment of the potential impact on employment that the current economic crisis will have on some manufacturing activities across Europe. The analysis is realised employing the concept of vertically integrated labour (Pasinetti 1973) which allows to account for the employment directly *and* indirectly involved in the production of final goods. The estimations are derived from Multi-Regional Input–Output tables to map the supply chain and to differentiate between the employment involved in the production of exported intermediate inputs and final goods. The results show that most of the employment involved in the production of final output of the activities studied in the paper is linked to international trade. Although Europe shows a high degree of productive links, there are important differences in the modality of insertion in the productive structure of European countries. Moreover, the impact on the level of employment due to the current economic crisis can be significant, affecting more than 1.3 million of people in Europe. These results are relevant to policy makers, who should consider carefully the high degree of linkages of the European economies when designing industrial policies and measure of support to the economy.

## Introduction

After approximately a decade from the global financial collapse, a new crisis, triggered by the Covid-19 pandemics, is shaking the global economy. The effects on the economy are likely to be prolonged, in what already appears to be the most severe economic downturn since the Great Depression (Gopinath 2020).

Most of the studies published so far that deal with the consequences of the current crisis are concerned with aggregate figures like GDP (IMF 2020) or with the direct sectoral repercussion (e.g. McKenzie 2020), neglecting the indirect impact that the economic lockdown imposed across countries and the loss in final demand will have on the supply chain. However, for a thorough assessment of the implications of an external shock on the productive system it is important to focus not only on the effects that it may have on a specific industry, but also the backward linkages of the supply chain. The relevance of Global Value Chains (GVC) and the role of trade in intermediate goods (Johnson and Noguera 2012; Kleinert 2003; Miroudot et al. 2009), impose a systemic consideration of the production processes.

The paper addresses these aspects by focusing on two research objectives: first, it maps the degree of productive integration of four productive activities in Europe, focusing on the generation of employment in the production of exported intermediate inputs and final goods. Second, the paper provides a preliminary assessment of the potential impact of the ongoing economic crisis on the level of employment in some manufacturing activities across Europe. To the best of our knowledge, this is the first study to assess the impact in these activities with a similar degree of detail at the European level. While the present research will not engage directly with forecasts regarding the impact of the current Covid-19 crisis on the degree of integration of the European supply chain, it offers useful elements to consider the possible propagation mechanisms, along the supply chain, of the Covid-19 crisis and to design effective industrial policies.

The analysis considers four key activities of the European manufacturing: Manufacture of Motor Vehicles; Manufacture of Other Transport Equipment; Manufacture of Computers, Electronics and Optical goods; Manufacture of Textiles,^1^ using the notion of subsystem (Sraffa 1960) and vertically integrated labour (Pasinetti 1973).

This approach enables to quantify the amount of employment that depends, directly and indirectly, on these four activities (henceforth, the term subsystems will be used to denote the focus on both direct and indirect relations of production). In particular, the empirical analysis will distinguish between the employment involved in the production of intermediate and final goods (for domestic and foreign consumption) and differentiate the impact among 27 European countries. Calculations are performed in a Multi-Regional Input–Output (MRIO) framework which allows to account for the role played by international trade (of intermediate and final products) in the production process.

What emerges from the empirical analysis is the predominance of foreign markets: most of the employment in these four subsystems can be associated to the production of exported goods (both in intermediate and final goods). Moreover, the multi-regional level of analysis allows to establish which countries are more exposed to the downturn in the level of activity due to a breakdown of the value chain, both in terms of production and final consumption. Then, the paper provides some preliminary estimates of the potential impact, in terms of jobs losses, of the current economic crisis in the four subsystems studied here. We find that the impact on the employment levels in these subsystems can be significant, and especially concentrated in the Automotive supply chain. These findings have important implications for economic policy. Industrial strategies and the recovery measures that will be put in place should take into consideration the high degree of productive linkages existing among European countries. The high reliance on exports implies that uncoordinated domestic measures alone may be insufficient in stimulating economic recovery. Finally, the paper argues that ideal economic policies should aim at reverting the disparity that has mounted in the last decades in Europe (Guarascio and Simonazzi 2016) in terms of productive capacity and technological catch up.

The paper is organised as follow, Sect. 2 presents the relevant literature and discusses it in light of our objectives. Section 3 describes in detail the methodology used for the calculation of vertically integrated labour. Section 4 presents the results of the empirical analysis and Sect. 5 discusses the main findings and some relevant policy issues.

## Literature review and discussion

The economic crisis induced by the Covid-19 pandemic is going to have a deep impact on economic activity (IMF 2020; Maliszewska et al. 2020). However, the negative shock will be heterogeneous across industries and countries since some manufacturing activities are more exposed to the current crisis than others. One of the aspects that characterises the current situation is the variety of disruptions channels which derive from both the supply and demand side. On the supply side, both physical distancing and economic lockdown had, although different across countries, a direct impact on the production processes of good and services, where the intensity of the productive disruptions is reinforced by the strong supply chain links. On the demand side, the drop in aggregate demand and the postponement of the purchase of durable goods are likely to have long-lasting effects on production (Baldwin and Tomiura 2020). These aspects are especially relevant for the four industries analysed in this paper. These activities represent 20.5% of total employment at the industry level in European manufacturing. Their importance is even more striking when considering the indirect relations of production. If we account also for the employed generated indirectly in the production of the final output of these industries, the share of employment amount to 35% of the total activated by manufacturing in Europe (see Sect. 4 for details). Another common aspect is that the four industries analysed in this paper have been classified as non-essential in most countries and had to partially or completely stop producing during the peak of the pandemic (Fana et al. 2020) which, in combination with the strong reduction in final demand that they are likely to suffer, may lead to a sizeable reduction in the employment levels. Lockdown measures had a great impact on the level of activity. Giammetti et al. (2020) estimate that the lockdown measures had an impact of approximately 52% of circulating value added. These findings are in line with those performed including a richer pool of countries, that estimate a reduction between 30 and 50% of the level of activity during the first lockdown (OECD 2020a).

When assessing the impact of an economic shock on the productive structure, the high level of interdependence of manufacturing activities in Europe, both in terms of productive linkages and as source of demand of final products (Eurofound 2019), imposes to focus not only on the direct, industry-level, relations of production, but also to acknowledge the indirect effects on the industries that provide intermediate inputs. Some researchers are already considering the possibility that the ongoing crisis may lead to a deep rearrangement of the international productive processes, fuelling the reshoring of western companies (e.g. see Seric and Winkler 2020; Strange 2020). This discussion links with the existing contributions that focus on the process of deglobalization and reshoring (for an overview see De Backer et al. 2016). While there is some evidence of reshoring in some activities in the last decade, this is not sufficient to constitute a reversal in the aggregate trend (Bailey and De Propris 2014; Kinkel 2014). In fact, reshoring is, so far, a limited phenomenon among European firms and mostly confined to companies that move back production from Asia but not from other European countries (Dachs et al. 2019). Another possibility that is being considered is that the supply chains will become more *regionalised* (Zhan et al. 2020). According to this view, instead of counting with strong global/intercontinental links, supply chains may be reoriented towards the exploitation of productive links in a certain region/continent. This latter scenario would be extremely relevant for the European context, where many countries are distributed in a relatively small geographical area. Independently from the evolution of the GVC reconfiguration that will follow the current economic crisis, it is paramount to examine the potential impact of the current economic crisis on the existing productive structure in the context of the high degree of productive integration existing in Europe. While the present research will not engage directly with forecasts regarding the impact of the current Covid-19 crisis on the degree of integration of the European supply chain, it offers useful elements to consider the possible propagation mechanisms, along the supply chain, of the Covid-19 crisis and to design effective industrial policies.

The importance that offshoring and trade in intermediate inputs play in the productive integration of Europe has been highlighted by the literature. For example, Marin (2006) shows the expansion of German and Austrian companies in Eastern European (EE) countries to offshore intermediate steps of production. Miroudot et al. (2009) stress that European countries have experienced a considerable increase in trade in intermediate goods at the beginning of the 2000s. More recently, Timmer et al. (2013) account for the generalised increase of employment activated by the production of intermediate inputs and the rise of imported intermediates versus domestically sourced intermediate inputs. Overall, these works provide precious insights about the general trends witnessed in the manufacturing sectors in Europe. None of these studies, however, provide a thorough mapping of the role of European trade in intermediates for the four activities considered in this paper.

For this reason, and in relation to our first objective, this paper maps the degree of integration in the four economic activities mentioned earlier. In particular, we are interested in understanding what type of employment (whether for the production of intermediate exported inputs or final goods) is activated by the four subsystems across European countries.

This analysis relates to the recent debate around the growth model to be implemented. As noted in the literature, *core* countries (e.g. Germany) underwent a reorganisation of their trade and manufacturing, with a shift in exports from Southern European (SE)^2^ countries towards China and an expansion of their industrial base in the Eastern periphery (Celi et al. 2019). The increasing international competition has been often employed as a justification to implement internal devaluation strategies, whose aim is to foster exports rather than favouring domestic demand (Uxó et al. 2014). This reconfiguration of the European Value Chains has been accompanied by different trends in sectoral specialisation which, in turn, shaped the dynamics of the occupational structures across European regions. As shown by Gräbner et al. (2020a, b) Germany is characterised by a higher share of exports in more complex goods than SE countries while Eastern European countries have benefitted from a structural upgrade (Stöllinger 2016). Hurley et al. (2019) show that in some EE countries like Poland the share of medium and high paid workers has increased more than at the European level while SE has been characterised by the increase in low-paid jobs. In this sense, Celi et al. (2020) report that, since 2010, SE countries have witnessed a boom in the number of firms in services like the Tourism industry, reducing the presence of manufacturing firms, while core countries experienced the opposite trend. Similar to these studies, our research contributes to the literature regarding the pattern of specialisation and job creation in Europe. By distinguishing between the employment embodied in the trade of intermediate and the employment that does not leave the domestic supply chain it is possible to provide a characterisation of the type of insertion of a certain country in the supply chain. For example, a high generation of employment associated to the trade in intermediate inputs indicates a high reliance on the decision of production taken by companies located in third countries.

With respect to the second objective, this paper provides a preliminary study about the potential impact of the ongoing economic downturn on the employment generated in these subsystems. At this stage, it is not easy to quantify the effect that the ongoing crisis will have in terms of jobs loss. The impact on employment will be largely influenced by the duration of the pandemic and by the response adopted by policy makers to support both the supply and demand side (see Sect. 4 for a detailed discussion). It is however possible to provide some estimates of the consequences that the current crisis could have on the industries directly involved in this analysis and on the industries taking part indirectly in the supply chain as provider of intermediate inputs. This analysis will consider the direct impact as well as the indirect effects. The relevance of considering the fall in the level of trade in intermediates following economic crises has been documented in different works (e.g. Alessandria et al. 2010) together with the necessity of accounting for the indirect spillovers of a fall in final demand on other countries (Bems et al. 2010). These aspects are especially valid for the activities studied in this paper, given the high participation intermediate suppliers and the high degree of outsourcing (e.g. see Sturgeon et al. 2008 on Automotive).

Overall, this analysis is essential to design effective economic policies. Industrial policies are coming back on the agenda (Wade 2012; Rullani et al. 2016) and, at least formally, are at the centre of the European strategy for the upcoming years (EC 2019). However, as it has been highlighted by some authors (Celi et al. 2018; Stöllinger 2016), the process of productive integration and participation in GVC has not benefitted equally all European countries. One of the risks of the ongoing economic crisis is that the existing polarisation will be deepened, similarly to what happened in the previous recent economic crises, and that given the existing productive structures SE countries will be the most affected (Gräbner et al. 2020a, b). Because of the productive heterogeneity and the high level of productive linkages of the European manufacturing, domestic uncoordinated policies may be insufficient to restore the employment level. In this sense, Portella-Carbó and Dejuan (2019) have showed that an uncoordinated expansionary fiscal policy at the European level may reinforce the productive disparities between core and peripheral areas in Europe. In theory, this crisis could be the opportunity to reopen the debate about the imbalances that have manifested in the Europe in the last decades (see Simonazzi et al. 2013). Along these lines, different contributions highlight the necessity of a collective response to the crisis. Coveri et al. (2020) advocate for a coordinated industrial policy that aims at reducing the productive asymmetries that have grown in Europe in the last decades, with a special attention at the vulnerability that derives from hyper specialisation in some segments of the supply chain. As it will be argued below, the concentration in some parts of the supply chain (e.g. in the production of exported intermediate inputs) may increase the degree of vulnerability of some countries. The need for coordinated monetary, fiscal and industrial policies is especially important in the current context, with an accentuated global crisis which affects both the demand and the supply side (Baldwin and Weder Di Mauro, 2020) and would need a considerable revision of the current inflexible policy framework (for a discussion see Celi et al. 2020; Pianta et al. 2020).

In order to promote coordinated productive policies, it is essential to have a clear picture of the productive links and their geographical distribution. This would provide valuable elements to design economic measures that take into consideration the specificities of the supply chains, at the domestic and European level. As an empirical exercise aimed at stating the importance of productive integration, we restrict our analysis to a selection of manufacturing industries. Although this choice does not allow to draw conclusions for the whole manufacturing sectors in Europe, it permits to focus on some specificities of the selected activities. The selection made in the paper considers the heterogeneity of industrial activities in that our four sectors differ in their contribution to overall EU value added, value of intermediate inputs and their employment share over total EU-27 employment. The four sectors are characterised by different levels of technological content, according to the classification proposed by Galindo-Rueda and Verger (2016), with Computer and Electronics being High Tech; Automotive being Medium–High Tech and Textiles being classified as Medium–Low Tech. According to this classification, Other transportation includes both High Tech (Air and Spacecrafts) and Medium–High Tech (Railroad and Ship building) activities.

## Methodology

In order to deal with our research objectives, this paper employs the notion of vertically integrated labour, based on the concept of vertically integrated sectors or subsystems (Pasinetti 1973). One of the major advantages of this approach is that it allows to deal with both direct *and* indirect relations of production. This means that it is possible to account not only for the direct impacts of a loss in activity in the specific industry under analysis, but to quantify also the (indirect) effects on the industries that provide intermediate inputs to a given industry (and on the industries that provide intermediate goods to the intermediate inputs industries and so on).^3^

Vertically integrated labour is calculated as follows. Using a Multi-Regional Input–Output (MRIO) framework, the vector of total output is equal to the sum of intermediate inputs produced and net output:

1 $$\mathbf{x}= \mathbf{Z}\mathbf{i}+\mathbf{y}$$

where **Z** represents the multi-regional transactions matrix composed by r * s sub-matrices. Each sub-matrix ${\mathbf{Z}}^{rs}$ on the main (*r* = *s*) diagonal represents the matrix of domestic transactions, and off-diagonal sub-matrices are the matrices of international transactions of intermediate inputs, i.e. imported inputs (see Miller and Blair 2009, Chapter 3). Each industry in column *j* in the submatrix ${\mathbf{Z}}^{rs}$ (dimension i * j) demands intermediate inputs from the industries indicated in rows *i*. The term **x** is the multi-regional vector^4^ of total output, **y** the multi-regional vector of net output (i.e. final demand) and **i** is the sum-vector of appropriate length. Expression (1) can be reformulated as:

2 $$\mathbf{x}= \mathbf{A}\mathbf{x}+\mathbf{y}$$

where **A** is the MRIO matrix of technical coefficients. Each technical coefficient (${\mathrm{a}}_{ij}^{rs}=\frac{{\mathrm{z}}_{ij}^{rs}}{{\mathrm{x}}_{j}^{s}}$) indicates input from industry *i* and country *r* demanded for the production process of sector *j* in country *s*. Following few manipulations, the vector of total output can be expressed as:

3 $$\mathbf{x}={\left(\mathbf{I}-\mathbf{A}\right)}^{-1}\mathbf{y}$$

where **I** is the identity matrix and ${\left(\mathbf{I}-\mathbf{A}\right)}^{-1}$ is the MRIO Leontief inverse matrix of dimension ri * sj. Pre-multiplying the right-hand side of Eq. (3) by the vector of direct labour coefficients (${\mathbf{a}}_{\mathrm{l}}^{\mathrm{T}}=\mathbf{l}{\widehat{\mathbf{x}}}^{-1}$, where **l** is the vector of employment by industry), we finally obtain the vector of vertically integrated labour as:

4 $${\mathbf{l}}_{vi}^{\mathrm{T}} ={{\mathbf{a}}_{\mathrm{l}}^{\mathrm{T}}\left(\mathbf{I}-\mathbf{A}\right)}^{-1}\widehat{\mathbf{y}}$$

where the vector of vertically integrate labour (${\mathbf{l}}_{vi}^{\mathrm{T}}$) represents the amount of total labour needed in each subsystem *j* in country *s* to satisfy the level of final demand. The focus on the subsystem, instead of industry level implies that the values in ${\mathbf{l}}_{vi}^{\mathrm{T}}$ represent the number of units of labour employed directly and indirectly (i.e. in the production, direct and indirect, of the intermediate inputs and so on) in each subsystem and country in the production of final output. The vector ${\mathbf{l}}_{vi}^{\mathrm{T}}$ in Eq. (4) is a concise indicator that does not differentiate between the employment generated domestically or in other countries, i.e. via the demand of imported inputs of production. As argued above, this is a crucial aspect to take into consideration in contemporary manufacturing activities, since a consistent part of the workforce is employed in the production of intermediate inputs that are then employed in different countries and industries.

To differentiate between the domestic and imported employment demanded by a given subsystem it is sufficient to employ the diagonalised vector of direct labour coefficients in Eq. (4), so that:

5 $${\mathbf{L}}_{vi} ={\widehat{{\mathbf{a}}_{\mathbf{l}}}\left(\mathbf{I}-\mathbf{A}\right)}^{-1}\widehat{\mathbf{y}}$$

By doing so, we obtain a matrix of vertically integrated labour (${\mathbf{L}}_{vi}$) where the cells of each column indicate the employment needed from the activity *i* and country *r* in the production of subsystem *j* in country *s*.

Data employed for the estimations of this paper are obtained from the World Input–Output Database (Timmer et al. 2015). This database provides MRIO tables that map the global economy with data for 43 countries (and one “rest of the world” region) and 56 industries organised at the 2 digit ISIC 4 level (Timmer et al. 2016). The estimations are based on the most recent year available in the database, 2014. Although it would have been ideal to have more updated data, this database provides a good approximation of the existing productive structure.

Note that this approach is based on physical units of labour that are embodied in the final production of goods which reflects the standard demand-driven orientation of the original Leontief model (Leontief 1944). Furthermore, another significant aspect of this type of analysis is that the variable of interest (i.e. vertically integrated labour) is expressed in physical terms as units of employment that are directly and indirectly employed in the production of a certain final output which represents a difference to other MRIO estimations based on value added (e.g. OECD Trade in Values Added database). By expressing the unit of interest in physical terms, this approach allows to focus on the technical features of the productive chain without being influenced by wage differentials across countries. In this way, only technical changes that affect the amount of labour employed in the production process are grasped by the vector (and matrix) of vertically integrated labour, being neutral to the evolution of unit labour costs.

## Results

This section presents the empirical results for a selection of manufacturing activities in Europe: Automotive, Other transportation, Computers and electronics and Textiles.

**Fig. 1:**
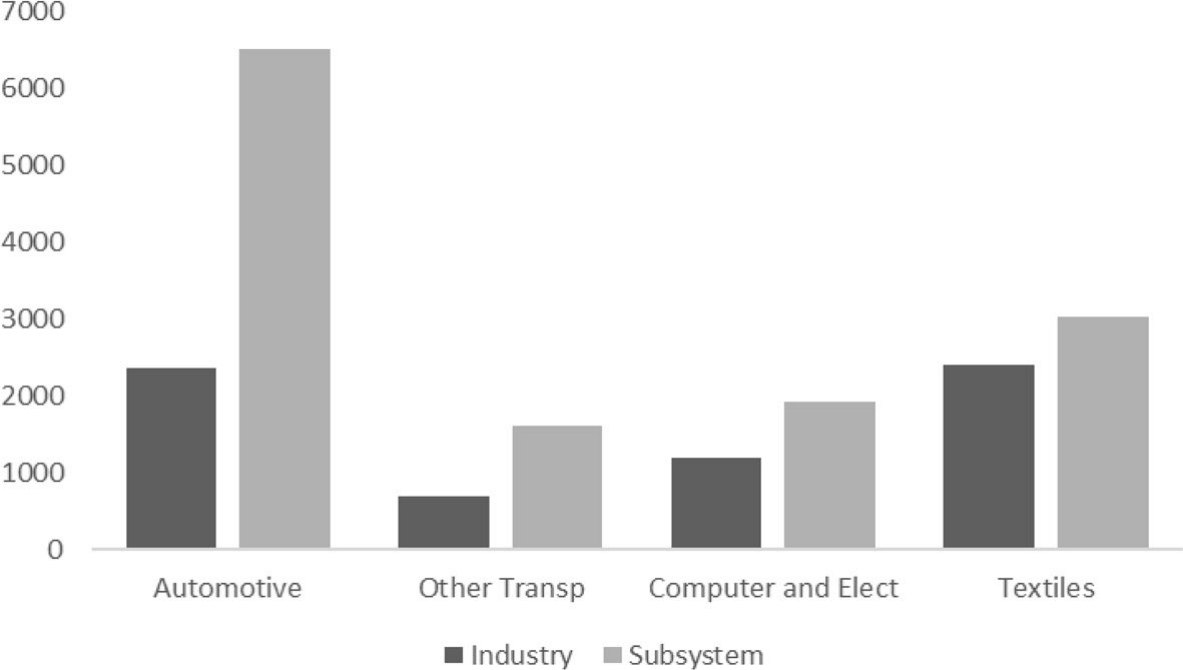
Number of labour units (thousands) employed at the direct-industry level and subsystem for the whole sample of European countries. Year 2014. Source: Author’s elaboration using the WIOD database

The first look at the results is represented by the estimation of the number of people employed by subsystem. Differently from the standard industry-level studies, the estimates at the subsystem level capture the number of workers employed in those industries that directly and indirectly participate in the production of final output in each subsystem. Figure 1 shows that all these activities involve a higher amount of labour at the subsystem level than they do at the direct (industry) level. The difference between industry and subsystem level is particularly marked for Automotive and Other Transportation, indicating the relevance of the backward linkages that the production of the final goods in these subsystems entail.

In order to account for the role played by intermediate inputs in the generation of employment, for each country and subsystem, we further split, from the matrix of vertically integrated labour ${\mathbf{L}}_{vi}$, the total amount of labour between the labour force that is generated by the foreign demand of intermediate inputs and the fraction involved (directly and indirectly) in the domestic production of final goods.

**Fig. 2:**
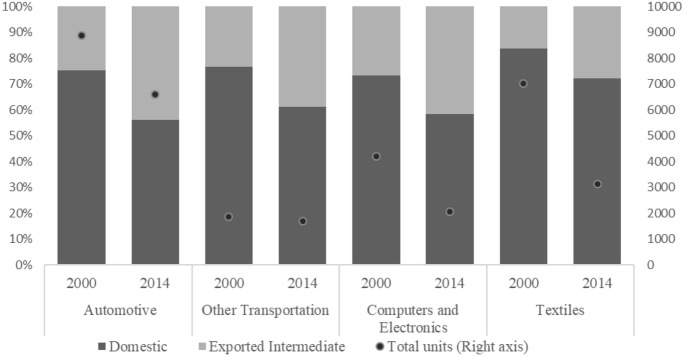
Share of employment involved in the production of exported intermediate inputs over total employment and units of employment (thousands) by subsystem in 2000 and 2014 for the whole sample of European countries. Source: Author’s elaboration using the WIOD database

Figure 2 illustrates the total amount of employment activated by these subsystems in 2000 and 2014 and differentiates between the participation of the employment embodied in the production of exported intermediates inputs and the labour that is embodied in the domestic production of final goods. First, it is worth noticing that the total European labour force involved in the four subsystems has diminished considerably between 2000 and 2014, with the only exception of the Other Transportation subsystem. This aspect reflects the process of downsizing of the manufacturing activities recorded at the beginning of the century which has been enhanced by the process of offshoring and the increase in the number of extra-European competitors (Stöllinger et al. 2013). Moreover, this reduction should not be imputed to the outsourcing process that took place at the industry level, since this figure does not refer exclusively to direct employment but also includes the indirect relations of production. Second, the share of labour involved in the production of exported intermediate goods and services grew remarkably between 2000 and 2014 among all the subsystems. Approximately 40% of the total European employment is activated by the trade in intermediate inputs showing the increasing fragmentation of the production processes within European economies.

For a more detailed assessment of the relative importance of these subsystems in terms of employment creation across European countries, the first four columns in Table 1 show the distribution of the total employment by subsystem across European countries while the last column represents the share of *total* employment of each country over total employment in Europe. A share of labour created in a certain subsystem that is higher than the share of total employment of the country at the European level (last column) indicates that the subsystem has a relatively higher relevance for the country compared to its average participation in European employment.

**Table 1:** Distribution of the vertically integrated labour across countries and subsystems (as % of the total European labour by subsystem) and share of each country’s total employment in Europe (last column). Source: Author’s calculations based on WIOD data

	Autom	Other Transp.	Comp and electronics	Textiles	Total
Austria	1.6	1.4	1.9	1.0	1.9
Belgium	1.7	1.3	1.6	1.4	2.0
Bulgaria	1.0	1.1	1.3	5.4	1.6
Cyprus	0.1	0.0	0.1	0.3	0.2
Czech Republic	5.8	2.8	4.3	2.0	2.3
Germany	33.8	26.2	27.7	9.4	19.0
Denmark	0.4	0.5	1.2	0.4	1.2
Spain	5.7	6.9	3.7	7.7	8.0
Estonia	0.1	0.2	0.4	0.5	0.3
Finland	0.4	0.9	2.4	0.6	1.1
France	6.9	15.9	7.2	6.3	12.1
Great Britain	7.6	10.2	9.6	4.2	13.6
Greece	0.5	0.4	0.7	2.3	1.8
Hungary	3.1	1.2	4.3	1.8	1.9
Ireland	0.4	0.3	1.5	0.4	0.8
Italy	8.4	13.1	8.2	20.3	10.8
Lithuania	0.2	0.2	0.4	1.5	0.6
Luxemburg	0.1	0.1	0.2	0.1	0.2
Latvia	0.1	0.3	0.3	0.5	0.4
Malta	0.0	0.0	0.1	0.1	0.1
Netherlands	2.1	3.2	4.0	1.6	3.9
Polonia	8.6	6.6	8.4	9.1	6.9
Portugal	1.2	0.8	1.5	7.2	2.0
Rumania	5.3	3.8	3.8	13.1	3.9
Slovakia	2.2	0.7	1.5	1.4	1.0
Slovenia	0.5	0.3	0.6	0.7	0.4
Sweden	2.0	1.7	3.2	0.7	2.1
Total	100.0	100.0	100.0	100.0	100.0

From this table it can be appreciated that the four subsystems are particularly relevant for Germany and the non-Baltic Eastern European (EE) countries.^5^ These countries record at least two subsystems whose share of employment is above the participation of the country in the total European employment. The only other case in the sample that count with at least two subsystems above the average is Italy (in the Other Transportation and Textiles subsystems). All other countries count one or zero subsystems whose share of employment is above their total participation in European employment. The importance of the network between Germany and non-Baltic EE countries in Automotive evidenced in the literature (e.g. Celi et al. 2018; IMF 2013) is also emerging from the table. These are the only countries that account for an absorption of labour in the Automotive subsystem that is substantially higher than their share in total European employment. Another interesting aspect is that in all SE countries Textile, the only Low-tech activity among the four analysed in this paper, is the most important subsystem in terms of output generation.

**Fig. 3:**
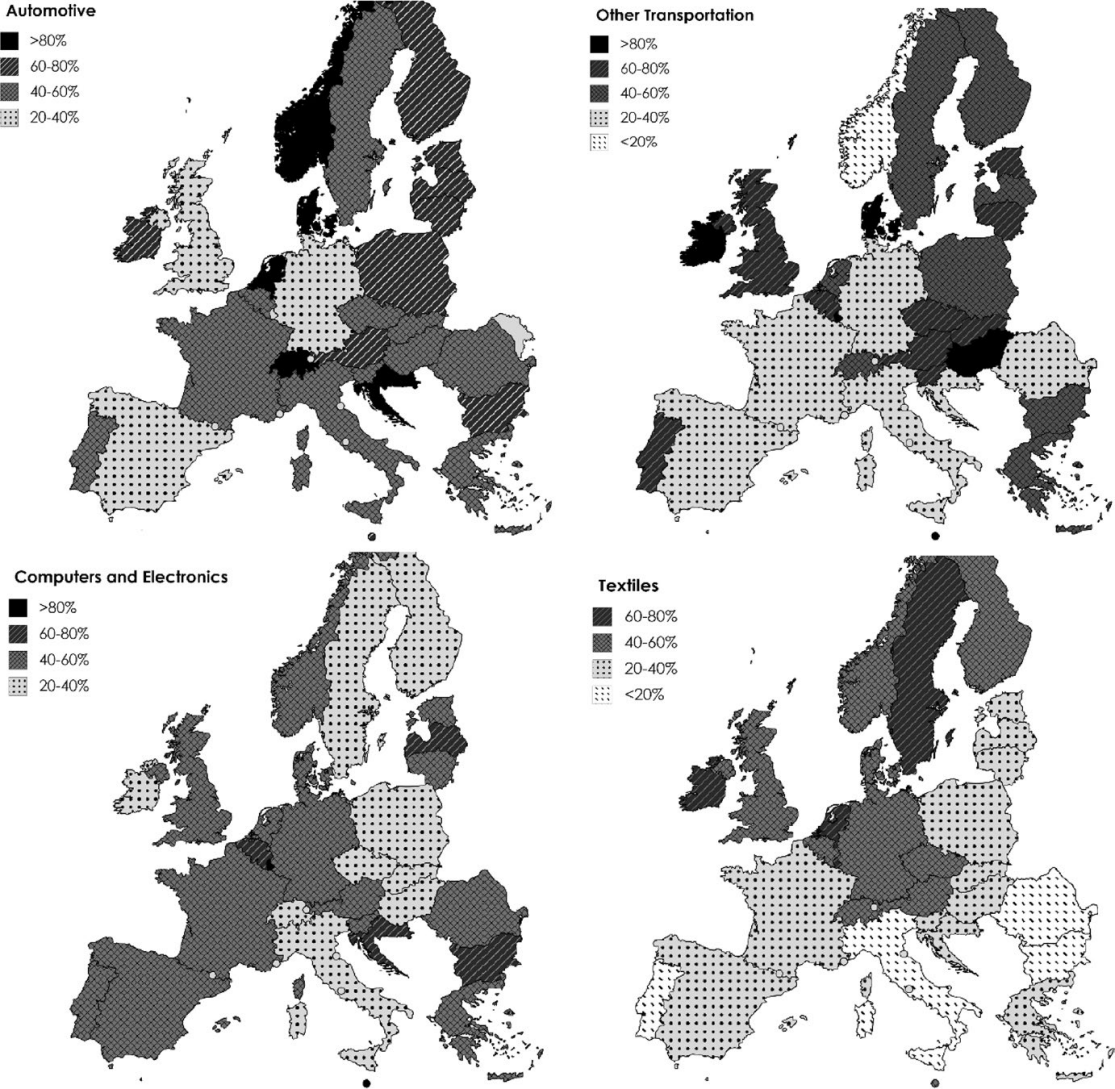
Share of labour involved in the production of exported intermediate inputs by subsystem. Year 2014. Source: Author’s elaboration using the WIOD database

Nevertheless, looking exclusively at the total generation of employment by subsystem provides only partial information. To the purpose of this paper it is important to distinguish between the type of employment generated since the incidence of intermediate inputs in the generation of employment is not uniform across countries. In fact, even in countries where a certain subsystem is particularly significant in the generation of employment (e.g. non-Baltic EE countries and Germany in Automotive), the participation in the GVC is not equivalent. Figure 3 displays, for each country and subsystem, the share of vertically integrated labour absorbed by the production of intermediate inputs that are then assembled in third countries. This data can be considered a proxy of dependence of a country on the final stages of production realised elsewhere. The higher this share, the higher the degree of dependence from other countries’ final production and their decisions of allocation of production in other countries. A pattern of specialisation based on the production of intermediate inputs which will be assembled and/or consumed elsewhere makes this production more vulnerable to external demand and shocks, out of control from national industrial and fiscal policies.

What emerges from this picture is that the Automotive subsystem accounts for the highest share of labour activated in intermediate inputs. In this case, only three countries (Germany, Spain, and Great Britain) record less than 40% of total labour involved in the production of exported intermediate goods. The level of involvement of labour in exported intermediate inputs is lower if we look at the other subsystems, although it is still very significant. In the case of Other Transportation and Computer and Electronics subsystems, only seven and nine countries respectively have less than 40% of total employment that is activated by the production of exported intermediate inputs. Textiles is the only subsystem where most of the countries account for less than 40% of labour that is activated by the trade in intermediates, denoting a higher role of the domestic supply chain in the generation of employment.

As to the geographical distribution, it appears that EE countries participate mostly as producers of intermediate goods and services which are then finally assembled in other countries. This is particularly valid for Automotive, with a share of exported intermediates between 60 and 80% in Poland and Czech Republic, and Other transportation subsystems. At the same time, the relative importance for this group of countries is lower in the manufacture of Computers and Electronics and Textile subsystem. On the other hand, the fact that countries like Germany record lower shares of labour involved in exported-intermediate inputs of production testifies the role of this country as final assembler/producer of final goods.

All in all, the empirical evidence reported confirms the strand of literature pointing to the strong productive integration characterising Europe as well as the structural dependence of EE countries on the production of intermediate inputs (especially in the Automotive and Other Transportation subsystems). SE countries show a lower participation in the production of exported intermediates. Note that this is especially valid for Textiles, which is also where three countries (Greece, Italy and Portugal) record a relative specialisation. This aspect indicates that in these countries the great majority of labour activated in this subsystem is maintained in the domestic supply chain.

By construction, all the employment that is not embodied in intermediate exported inputs, is devoted to the domestic production of final goods. This amount of labour force can, in turn, be divided between the amount of employment that is needed to produce final goods for domestic consumption and final goods that are exported to third countries. As noted by Strange (2020, p. 462) the interest about the reconfiguration of GVC should not draw the attention away from the fact that many products (and jobs) are strongly dependent from the production of final exported goods and services. Table 2 indicates that over the four subsystems, between 49% (Textiles) and 69% (Other Transportation) of the employment that is not generated in the production of exported intermediate inputs, is activated by the production of final goods that are exported. These figures prove the high degree of dependency of European labour on international trade. Therefore, it can be concluded that, in all the subsystems analysed in this paper, the great majority of the labour force is involved in international trade, whether in the production of goods and services that are exported as intermediate inputs or as final goods. This aspect should be considered carefully by policymakers since, given the high degree of dependence on external markets, country-level and uncoordinated policies risk to have a dispersed and limited effect.

**Table 2:** The role of exports of final goods in employment creation by subsystem. Year 2014. Source: Author’s elaboration using the WIOD database

	Automotive	Other transport	Comp. and electronics	Textiles
No. of employed (thousands) for exported production of final goods	2279	667	668	1084
% of employment of exported final production over the total number of workers employed in the production of final goods (consumed domestically + exported)	61.5	68.5	60.6	49.2
% employment of exported final production over total employment in the subsystem	35.1	42.8	37.7	36.5

Finally, given the strong productive linkages that exist within these subsystems, it is worth asking about the potential impact of the ongoing crisis in terms of potential jobs loss and be aware of the risk of possible deepening of the disparities that could follow an economic downturn. Although we must be cautious when drawing parallelisms between past and current events, recent experience has proven that the economic recessions in the European arena can foster divergent trends across countries. In this sense, the response to the Global Financial crisis (2008–2009) and the debt crisis (2011–2012) is paradigmatic. Even though the drop of demand affected all countries in Europe, the harsh austerity policies that were implemented in SE countries fuelled divergent trends in Europe. Employing Input–Output analysis, Garbellini et al. (2014) demonstrate that while austerity measures impacted the level of activity in the Eurozone, some core countries (e.g. Germany, Austria) responded to this shock looking outward the European space, replacing the drop in demand of the European periphery with more intense relationships with BRIC countries. These tendencies contributed to the divergent path of growth of SE countries compared to core countries. The consolidation of the divergent trends in Europe and the negative role of austerity policies has been shown also by Kohler and Stockhammer (2020), while Gräbner et al. (2020a, b) show that income, unemployment and technological capabilities between core and periphery have diverged considerably after 2008.

**Fig. 4:**
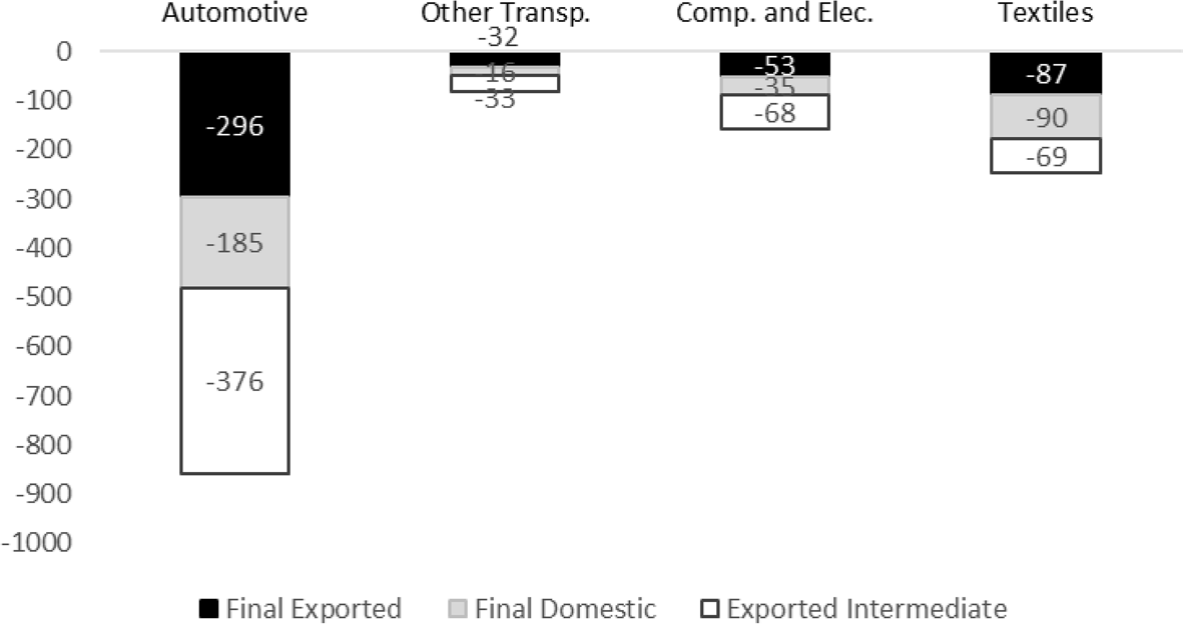
Estimated loss of units (thousands) of employment by subsystem in 2020. Source: Author's elaboration using the WIOD database (year = 2014) and Baker and McKenzie (2020) forecast for the fall in global production of each sector

In the present analysis, we build the estimations of the possible repercussions on employment using McKenzie (2020) forecast that, for 2020 reports a drop of 13% in the global production of the Automotive sector, 8% for Computer and Electronics and Textiles and 5% for Other Transportation. At this stage, it is still not possible to provide a precise analysis of the extent of the shock on the existing productive structures. Assuming that the fall in the level of final demand by industry will be distributed evenly across European countries, Fig. 4 shows the impact that the economic recession may have in terms of employment. Note that the assumption that the impact of the drop in demand will be equally distributed across European countries is a strong one. Much of the impact in terms of job loss will depend on the consequences that the Covid-19 crisis will have in terms of activity and on the measures put in place by the governments and international institutions to contrast the crisis. Moreover, most countries have implemented job retention schemes to support more vulnerable workers reducing at least in the very short terms the impact in terms of job loss. However, these measures differ in their extent (duration, selection criteria etc.) and are meant to be temporary (OECD 2020b). This implies that the impact on the employment due to the Covid-19 crisis may not be fully tangible in the short run but will need a period to adjust. Furthermore, the fiscal stimuli that will be introduced in the upcoming years may reduce the negative impact in terms of employment and countries that have a more active role in industrial policies may react more effectively than others. At the same time, changes in the patterns in final demand may favour some countries rather than others. For example, the growth of digitalisation and automation may boost those activities related to the Computers and Electronic subsystem, compared to more traditional activities such as Textiles. Finally, the modality of insertion in the productive chain may place countries in different positions when designing industrial policies. Countries that are mainly providers of intermediate inputs may be in a more fragile position in designing expansive measures compared to other countries (that lead the production process). Acknowledging these aspects, as an empirical exercise, we believe that it is still useful to offer a preliminary scenario of the potential impact of the crisis which takes into account direct and indirect relation of production as well as the international linkages of production. This exercise allows to visualise the potential degree of pervasiveness of the economic downturns on the European economy. Moreover, the analysis proposed in Fig. 4 distinguishes between jobs involved directly in the production of final goods (domestically consumed or exported) and those involved indirectly in the production of intermediate goods.

Over the four subsystems 1.3 million people can be affected by the drop in the level of activity. The impact is expected to be particularly marked in the Automotive subsystem, as a result of the high amount of labour that it generates in Europe (see Fig. 2) and the expectation of a more accentuated reduction of output compared to the other industries. Given the relatively higher importance of the German-non-Baltic EE network (Table 1), it is likely that these countries would be more affected by the downturn. In EE countries this impact will affect predominantly the activities that provide exported intermediate inputs, while in Germany the repercussions would be more evident in the activities involved in the domestic production of final goods. Textiles is expected to be the second most hit subsystem. In this case, the effects on employment are likely to be more localised, since the employment in this subsystem is less dependent on the exports of intermediate inputs compared to the others. Given the importance of this subsystem (see Table 1), SE countries are more exposed to the fall in final demand in this subsystem.

## Conclusions

The Covid-19 crisis is going to have deep repercussions on the European economies. This paper provides a preliminary assessment of the consequences that it may have in terms of jobs loss in four subsystems which employ (directly and indirectly) more that 13 million of workers at the European level.

A first aspect that emerges from this analysis is the high degree of dependence of employment on foreign demand via the export of intermediate inputs or final goods. Around two thirds of total employment in these subsystems is generated in the production of goods and services that are exported (as intermediate inputs or final goods). The impact on jobs generated by these subsystems can potentially be considerable, involving more than 1.3 million of people in Europe. The effects of the crisis are likely to be deeper in the Automotive subsystem, which is also the subsystem which accounts for the most developed network of indirect productive relations.

One crucial consequence of the trade in intermediate in the last decades is that an increasing share of employment is linked to the decisions taken by companies located elsewhere, which makes employment more vulnerable to decisions taken in third countries. Despite the growth in intermediate is a generalised trend, the intensity of production of intermediate inputs is not uniform across countries. What emerges from our study is a divide between Germany, EE and SE countries. In particular, Germany is specialised in more technologically advanced subsystems. In this country, the amount of employment generated by these subsystems is considerable and it is mostly concentrated in the domestic supply chain, as testified by the low share of employment embodied in the exports of intermediate inputs. The four subsystems analysed in this paper are also very important for the EE block. However, their participation in the productive chain is at odds with respect to Germany. In fact, these countries contribute mainly as providers of exported intermediate inputs of production which puts them in a more fragile (dependent) position in the production network as they are highly reliant on the investment decisions taken in other countries. Finally, these subsystems are relatively less important for the generation of employment in SE countries. Here, the most relevant subsystem is Textiles. Moreover, in this case, the productive network is more domestic centred since the great majority of employment (direct and indirect) activated by this subsystem is embodied in the domestic production of final goods.

In light of these results, we can advance some considerations regarding the future of European production networks. It is possible that the Covid-19 crisis will contribute to reshape the GVC. However, it is important to be cautious when tackling the hypothesis that a significant process of reshoring within Europe will take place soon. Unless there will be new differentiated and prolonged production lockdown measures or a radical institutional reconfiguration, it is reasonable to expect that the strong dependence on external markets will persist. European countries will still be highly dependent on external markets, at least in the short run. Moreover, it is not to exclude that the process of offshoring may intensify. Firms may be willing to exploit even more the wage differentials (which, in a context of high unemployment will possibly be maintained) pursuing further relocation of production in third countries to reinforce their profitability. This process may also be facilitated by geographical proximity and by the European infrastructure which plays a relevant role in facilitating productive integration (Landesmann and Stöllinger 2019).

The evidence provided here shows that the impact on employment of the COVID crisis is going to be severe and spread across countries. As a consequence, the measures that will be implemented by policymakers should consider the existing productive relationships across European supply chains. National policy measures are undoubtfully welcome in order to restore the level of activity and employment. However, the high dependence on foreign markets, via the exports of intermediate inputs and final goods, indicates that domestic fiscal expansion alone may be insufficient to restore employment levels, since most of the employment is dependent on external demand. Previous episodes have proven that economic crises can boost divergent trends in the European arena. Given the possibility that regional chains will be favoured compared to GVC (Zhan et al. 2020), it is crucial that policy makers in the EU design coordinated policy measures across countries in line with what has been proposed elsewhere (e.g. Coveri et al. 2020; Gräbner et al. 2020a, b). Moreover, industrial policies should be implemented, conceiving an evolving context and be concerned with the modification of the existing productive configuration (Pianta 2015). Since an undifferentiated fiscal stimulus would not affect evenly European countries, it is necessary to implement catching-up measures aimed at the conversion and upgrading of the productive structure of the peripheral areas of Europe that do not benefit from the *existing* specialisation pattern. This, of course, would imply the deep reconsideration of the stringent European regulations that have so far impeded the development of such policies (Pianta et al. 2020). There is no doubt that the current crisis will impact on the European economies. What is contended today is the response that institutions will be able to put in place. Given the primary role that the public sphere is expected to have in this picture, this new context may also allow to tackle pending crucial aspects regarding productive integration in Europe.

## Data Availability

The authors can provide access to data and material.

## Footnotes

1 For simplicity, hereafter these activities will be referred to as, respectively, Automotive; Other Transportation, Computer and Electronics, and Textiles.

2 Greece, Italy, Portugal and Spain.

3 For an illustration of this point see Pasinetti (Pasinetti, 1977, Chapter 4).

4 Lower-case bold letters denote vectors and upper-case bold letters matrices. All vectors are column vectors unless explicitly denoted by the superscript ^T^. The superscript ^ refers to a diagonalised vector.

5 Non-Baltic Easter European countries: Bulgaria, Czech Republic, Slovenia, Slovakia, Hungary, Poland and Romania.
